# Modified Polysulfone Nanofibers for the Extraction and Preconcentration of Lead from Aqueous Solutions

**DOI:** 10.3390/polym15143086

**Published:** 2023-07-18

**Authors:** Jessica Acuña-Nicolás, Tanese Montesinos-Vázquez, Irma Pérez-Silva, Carlos A. Galán-Vidal, Israel S. Ibarra, M. Elena Páez-Hernández

**Affiliations:** Laboratorio 2, Área Académica de Química, Universidad Autónoma del Estado de Hidalgo, Carretera Pachuca-Tulancingo Km. 4.5, Mineral de la Reforma 42184, Mexico; ac339710@uaeh.edu.mx (J.A.-N.); mo233519@uaeh.edu.mx (T.M.-V.); iperez@uaeh.edu.mx (I.P.-S.); galanv@uaeh.edu.mx (C.A.G.-V.); israel_ibarra@uaeh.edu.mx (I.S.I.)

**Keywords:** ion adsorption, separation, water treatment

## Abstract

Since lead is a highly toxic metal, it is necessary to detect its presence in different samples; unfortunately, analysis can be complicated if the samples contain concentrations below the detection limit of conventional analytical techniques. Solid phase extraction is a technique that allows the carrying out of a pre-concentration process and thus makes it easy to quantify analytes. This work studied the efficiency of sorption and preconcentration of lead utilizing polysulfone (PSf) fibers grafted with acrylic acid (AA). The best conditions for Pb(II) extraction were: pH 5, 0.1 mol L^−1^ of ionic strength, and 40 mg of sorbent (70% of removal). The sorbed Pb(II) was pre-concentrated by using an HNO_3_ solution and quantified using flame atomic absorption spectrometry. The described procedure was used to obtain a correlation curve between initial concentrations and those obtained after the preconcentration process. This curve and the developed methodology were applied to the determination of Pb(II) concentration in a water sample contained in a handmade glazed clay vessel. With the implementation of the developed method, it was possible to pre-concentrate and determine a leached Pb(II) concentration of 258 µg L^−1^.

## 1. Introduction

According to the United States Environmental Protection Agency, lead, along with other heavy metals, constitutes a health hazard because the population might be exposed to these contaminants from different sources such as cosmetics, spices, traditional or handmade ceramic ware, and contaminated drinking water [1].

Drinking water can be contaminated with lead when the taps or pipes that carry water to homes are made of this metal, or lead solder is used [2]. In particular, in the case of traditional pottery, lead (in the form of lead oxide) is used to give the clay pieces a shine and a waterproof finish [3]. This lead is leached when the pottery comes into contact with liquids or foods with acidic pH values. In a study by Diaz-Ruiz et al., [4] it was found that the amount of lead leached from glazed pottery ranged from 50 to 400 μg L^−1^, a concentration that had to be determined using a graphite furnace. The levels reached are noteworthy since the “action level” set by the Environmental Protection Agency is 15 μg L^−1^ [5]; in contrast, the WHO and the European Union maintain a minimum quality requirement for drinking water at 10 μg L^−1^ concentration at least until 2036, after which the 5 μg L^−1^ minimum will be applied [6].

As a result of these recommended levels of lead in water, its determination must be made by using sensitive techniques such as inductively coupled plasma mass spectrometry (ICPMS) or Graphite Furnace Atomic Absorption Spectrometry (GFAAS), which require considerable economic investment for their purchase and maintenance, or Differential Pulse Anodic Stripping Voltammetry (DPASV), which is a technique that requires greater specialization on the part of the analyst [7].

Although flame atomic absorption spectrometry (FAAS) is widely used, it has the disadvantage of not allowing the determination of lead at levels close to or below environmental standards. Thus, to facilitate lead analysis with FAAS due to its ease of operation and its attractive cost–benefit ratio, various investigations have focused on the development and use of materials that allow lead to be pre-concentrated in such a way that even low concentrations can be determined using this technique. Already known processes such as dispersive liquid–liquid microextraction have been adapted by using a smart solvent and adding 8-Hydroxyquinoline as a complexing agent [8], allowing the determination of lead present in lake water in concentrations ranging from 2.44 to 60.3 μg L^−1^, depending on the lake and the sampling season. In another work, a carbonaceous material made from sepiolite and glucose modified with 2-(5-Bromo-2-pyridylazo)-5-(diethylamino) phenol was used for the analysis of solutions of even 7 μg L^−1^, which after being preconcentrated with the developed material, could be determined using FAAS [9].

It is relevant to highlight that polymeric materials such as Amberlyst 15, a strong ion-exe resin with sulfonic acid groups, were used for the determination of lead in river water (0.070 μg mL^−1^) [10]. In the same way, Amberlite resin XAD-4 functionalized with 2,2,6-pyridine carboxaldehyde was used for determination of lead with FAAS coupled to a flow injection system, reaching a detection limit of 2.19 μg L^−1^ [11].

On the other hand, various researchers have opted for synthesizing new polymers that permit the determination of lead by employing a combination of dispersive solid-phase extraction (SPE) and FAAS. With the synthesis of poly 3-hydroxy butyrate-based comb-type amphiphilic copolymer containing vinyl benzyl tri-ethyl ammonium chloride [12], it was possible to determine a lead level of 1.3 and 8.9 μg L^−1^ in river and wastewater, respectively. Cellulose filters (nitrate and acetate) have also been used for separating lead after its complexation with 1,2,5,8-tetrahydroxyanthraquinone. The membrane loaded with the metal complex was dissolved for lead analysis with FAAS, obtaining, in the case of seawater, a content of 6.2 μg L^−1^ [13].

Polymeric fibers have also been utilized to remove lead mainly because they have a larger surface area and greater mechanical stability than other polymeric configurations. Thus, polymeric fibers manufactured by electrospinning made from unmodified polyvinyl alcohol (PVA) and modified with a biomaterial (*Malva sylestris* seeds) have reached a lead adsorption capacity of 444.2 mg g^−1^ and 588.2 mg g^−1^, respectively [14]. Additionally, fibers with an average diameter of 458 nm made of polyacrylonitrile modified with magnetite allowed the extraction of lead from aqueous solutions [15] with a maximum capacity of 156 mg g^−1^. Lead adsorption efficiency with these modified fibers is close to 95%, which is much higher than for other metals such as zinc and manganese; however, copper extraction is very close, reaching 80%. To have a more robust material, Pan et al. [16] prepared composite fibers of polyethylene with alginate modified with graphene oxide, with which lead removal of 386.5 mg g^−1^ was achieved through a process in which chemisorption plays a leading role. The material, which can be reused for at least 10 cycles, also extracts copper significantly (about 80%), although lead is completely extracted.

Currently, it is well known that modification of polymers is a necessary task to achieve the better extraction or removal of the species of interest, impacting its preconcentration. Such is the case of modification by grafting. Grafting usually refers to superficial modification or functionalization intending to confer specific properties without changing the macroscopic characteristics of the material. This surface modification has many advantages, as the added compound is retained covalently; various monomers can be used, and the resulting material is stable [17,18].

The grafting methods can be classified into three types: grafting from, in which polymeric chains are added on the surface of the material; grafting to, when a monomer is added and reacts on the active sites on the surface of the material; and grafting through, in which copolymerization with a monomer is carried out on a macromonomer [18,19].

Polymerization by free radicals is within the grafting from category, and can produce redox reactions. In this process, an initiator forms the radicals on the surface, and reacts with the monomer to form polymer chains. Ammonium persulfate and metabisulfite salts can be used as initiators. Persulfate is a thermal initiator, but in the presence of metabisulfite, mild conditions allow the grafting process (room temperature and aqueous medium) to obtain high yields and polymers with high molecular weight [19,20].

Grafted polymers have been used as sorbents for contaminants because functional groups such as amines, carboxylates, and sulfonates, among others, are attached to their surface. Some of these materials allow desorption and pre-concentration of heavy metals; thus, quantification of low concentrations (ng L^−1^ or µg L^−1^) can be performed using FAAS [21,22,23,24,25].

Following the above, in this work, we propose the use of polymeric fibers easily manufactured through blow spinning and modified by grafting for the analysis of lead at low concentrations with flame atomization atomic absorption spectroscopy. The variables that affect the elaboration of the material in its different phases and the extraction and re-extraction stages (which in total constitute the preconcentration process) of Pb(II) were studied, as well as the use of the complete process for the determination of leached lead in water that was contained in a handmade glazed clay vessel.

## 2. Materials and Methods

### 2.1. Reagents and Instruments

Polysulfone (PSf, M_n_ = 26,000 g∙mol^−1^), acrylic acid (AA, 99%) and potassium nitrate (KNO_3_, 99%) were purchased from Aldrich. Alizarin red S (C_14_H_7_NaO_7_S) and sodium hydroxide (NaOH, 98%) were obtained from Sigma-Aldrich (Saint Louis, MO, USA). Potassium metabisulfite (K_2_S_2_O_5_, 98%) and toluidine blue (TBO, C_15_H_16_N_3_SCl) were purchased from Sigma (Saint Louis, MO, USA). Chloroform (CHCl_3_, 99.9%), acetone (C_3_H_6_O, 99.9%), lead nitrate (Pb(NO_3_)_2_, 99.3%), nitric acid (HNO_3_, 69%), zinc nitrate (Zn(NO_3_)_2_), ethylenediaminetetraacetic acid disodium salt dihydrate (Na_2_EDTA·2H_2_O) (C_10_H_14_N_2_O_8_Na_2_·2H_2_O, 99%) were obtained from J.T. Baker (Radnor, PA, USA). Nickel nitrate (Ni(NO_3_)_2_), copper nitrate (Cu(NO_3_)_2_), and cadmium nitrate (Cd(NO_3_)_2_) were acquired from Meyer (Mexico City, Mexico). Ammonium persulfate ((NH_4_)_2_S_2_O_8_) was obtained from Merck (Darmstadt, Germany). Ethanol (70%) for general use (Ethyl alcohol denatured) was used without further purification. All aqueous solutions were prepared using deionized water with an 18.2 MΩ∙cm resistivity (Milli-Q Academic), Millipore, Bedford, MA, USA).

The concentration of metal ions was determined using flame atomic absorption with a Varian SpectrAA 880 spectrometer (Varian, Inc., Mulgrave, Australia); an Atomic absorption spectrometer Agilent 240 FS with GTA120 Graphite Tube Atomizer (Agilent Technologies, Mulgrave, Australia) was used to compare initial metal concentrations below 1 mg L^−1^. UV–Vis analyses were performed with a Perkin-Elmer Lambda 40 spectrophotometer (PerkinElmer Inc., Waltham, MA, USA) using a quartz cuvette with a path length of 10 millimeters.

### 2.2. Preparation of Polymeric Fibers

The polymer solution used for fiber manufacture was prepared by stirring for 4 h (at 25 °C) the corresponding amount of PSf and a binary mixture of chloroform and acetone in a ratio 4:1 (*v*:*v*), thus obtaining a homogeneous solution of known concentration (%*w*/*v*). This solution was used for fiber fabrication with the blow spinning technique [26]. This process was performed with a commercial airbrush with a 0.8 mm diameter nozzle connected to an air compressor (working pressure: 40 psi). The fibers were collected on an aluminum-coated cylinder attached to a rotary agitator (8 rpm). The aluminum foil with the collected fibers was dried at room temperature for 12 h and immersed in ethanol to easily remove the fibers.

In the process of making the polymeric fibers, the distance between the airbrush and the collector (10, 15 cm) and the PSf concentration (8, 9, 10, and 11% (*w*/*v*)) were evaluated. The appropriate airbrush–collector distance was selected considering the morphology of the fibers (number of fibers and nodules) visualized with a ProScope HR digital microscope with a 400× amplifying lens.

To select the best concentration of PSf, the alizarin red S extraction was evaluated. This dye was selected because it is easily retained in a wide variety of materials due to aromatic and electrostatic interactions [27]; thus, interaction with PSf is also feasible. The extraction of dye was carried out in batch systems with 10 mg of PSf fiber and 5 mL of an aqueous solution of 20 mg L^−1^ alizarin red S, which remained in stirring for 12 h. The sorption capacity of the fibers was determined by quantifying the concentration of dye before and after contact with the PSf fibers using UV–Vis spectroscopy at 423 nm.

### 2.3. Grafting of Acrylic Acid (AA) on PSf Fibers

The grafting reaction starts with the formation of free radicals due to the reaction of metabisulfite and persulfate. This generates radicals on the polysulfone surface which react with acrylic acid to form a covalent bond. Free radicals react with acrylic acid to form polymer chains on the surface of polysulfone [20].

Before the grafting process, fibers were cut to approximately an area of 25 mm^2^ to enhance the contact with the solutions employed in the process. Subsequently, a known amount of PSf fiber was left in contact with the ammonium persulfate/potassium metabisulfite initiator solution for 20 min [28]. After this time, an AA solution of known concentration was added, and the mixture was magnetically stirred for 100 min; in both steps, the temperature was controlled. Finally, the grafted fibers (PSf-AA) were washed with deionized water and dried at room temperature for 12 h.

The grafting process was studied using a 2^3 factorial experimental design. The concentration of the initiator solution (0.015 and 0.03 mol L^−1^), temperature (20 and 55 °C), and AA concentration (1 and 1.6 mol L^−1^) were evaluated. The number of carbonyl groups determined using TBO extraction was considered as a response, since this cationic dye can interact electrostatically with the carboxylates present [29]. The extraction process was carried out in batch configuration by adding 10 mL of 20 mg L^−1^ TBO to a sample of 30 mg of fiber to allow its interaction for 12 h with constant stirring at 200 rpm. The absorbance difference of the aqueous solution before and after extraction allows quantification of the number of carbonyl groups when analyzed at a wavelength of 630 nm by UV–Vis spectroscopy.

To corroborate the grafting of AA in the fiber of PSf, Fourier transform infrared spectra of (FTIR) were performed with a spectrophotometer Perkin Elmer System 2000 (PerkinElmer Inc., Waltham, MA, USA). In the same way, polysulfone fibers without AA were analyzed. The surface morphologies of the PSf-AA and PSf fibers were examined with a JEOL IT 300 scanning electron microscope with ×200 magnification and coupled with X-ray energy-dispersive spectroscopy (EDS) (JEOL Ltd, Akishima, Japan); scanning electron micrographs taken at a voltage of 10 kV.

To estimate the amount of AA grafted onto the PSf fibers, grafting density and grafting yield were calculated by measuring weight changes before and after the grafting process with an AD-6 Ultra Microbalance (Perkin Elmer) (accuracy of 0.1 mg). With the weight data and Equations (1) and (2), the graft density (*GD*) [30] and graft yield (%*GY*) [31] were calculated, respectively.

(1) $$GD=\frac{W_{f}-W_{i}}{W_{i}},$$

(2) $$\%GY=\left[\frac{W_{f}-W_{i}}{W_{i}}\right]\times \;100$$

where *W_f_* is the final weight of the PSf-AA fiber and *W_i_* is the initial weight of the PSf fiber.

**Figure 1:**
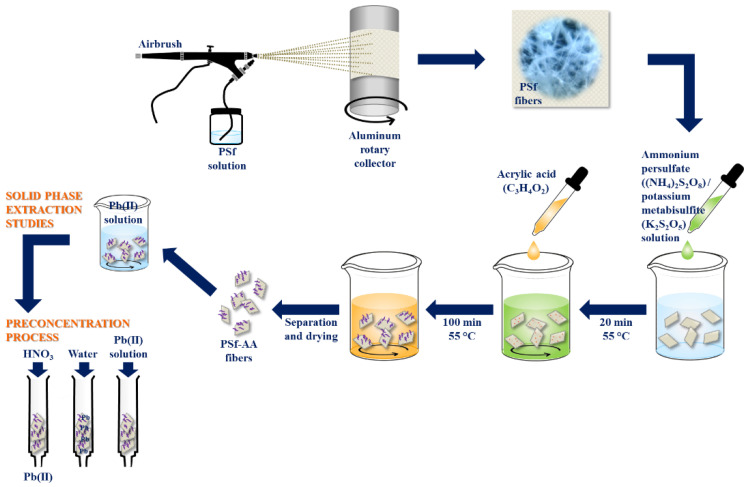
Schematic illustration for the PSf and PSf-AA fibers preparation.

A schematic illustration of the fiber preparation and grafted process is shown in Figure 1.

### 2.4. Pb(II) Preconcentration with PSf-AA Fibers

Prior to the preconcentration process, the Pb(II) extraction was studied to find the best conditions that allow the maximum sorption and, therefore, recover the most Pb(II) ions at a new aqueous solution

#### 2.4.1. Pb(II) Extraction Process

For the extraction experiments, a certain amount of PSf-AA fiber was weighed and mixed with a Pb(II) solution of 10 mg L^−1^ and stirred for a certain time (batch process). After this, the fibers were separated from the aqueous solution, which was analyzed with atomic absorption to determine the residual Pb(II). Different variables were evaluated through a factorial design of 2^4 to find the best conditions for the Pb(II) extraction process. The selected variables were pH (5 and 6), amount of adsorbent (10 and 20 mg), contact time (20 and 90 min), and ionic strength (1 × 10^−3^ and 0.1 mol L^−1^, adjusted with KNO_3_); all of them commonly affect the removal of metal ions [32].

To determine the sorption capacity of the PSf-AA fibers, the concentration of Pb(II) was evaluated from 1 to 100 mgL^−1^ following the best experimental conditions found in previous experiments.

#### 2.4.2. Effect of Interferents on Pb(II) Extraction

To evaluate the selectivity of PSf-AA fibers, common metal ions reported in studies of interferences in the sorption of Pb(II) were selected: Ni(II), Zn(II), Cu(II), and Cd(II) [32,33]. For this process, 5 mL of binary mixtures (Interferent: Pb(II)) were prepared in molar proportions 2:1, 1:1, and 1.5:1, using the extraction conditions in batch systems determined in the previous section.

#### 2.4.3. Re-Extraction of Pb(II)

After the extraction process, fibers were washed with deionized water. After being separated from the aqueous phase, they were placed again in batch systems with 5 mL of the selected eluent (Na_2_EDTA and HNO_3_). The mixture was stirred for 5 min, and after recovering the aqueous solution, it was analyzed with atomic absorption spectroscopy.

#### 2.4.4. Preconcentration Process

In this study, a column system was used instead of the batch system, since large volumes of initial solution could be used to achieve a better Pb(II) preconcentration. This also avoids fiber agglomeration and poor contact between fibers and the Pb(II) solution. For this reason, the volume of the initial solution and the amount of fiber was evaluated using the pH, and ionic strength determined in batch process studies.

The column process was completed in two stages: Initially, a certain amount of PSf-AA fiber (30, 40, and 50 mg of fiber) was packed in an insulin syringe without a needle, and then a certain volume of the Pb(II) solution (50 to 200 mL of 1 mg L^−1^ Pb(II) was passed through at 5 mL min^−1^ via flow pumping with a Cole Palmer Masterflex^®^ L/S model 7519-06 (Cole Palmer Instrument Company, Vernon Hills, USA) peristaltic pump. Before extraction, the Pb(II) solution was adjusted to the conditions (pH, ionic strength) established in the extraction experiments in batch configuration.

After the extraction process, the packed fibers were washed with deionized water. Finally, 5 mL of an aqueous solution of 1 mol L^−1^ HNO_3_ concentration was passed through to elute the Pb (II) retained in the PSf-AA fibers. 

To determine the minimum concentration of Pb (II) that can be quantified with the developed procedure and to evaluate the matrix effect, a correlation curve was made with the results obtained after applying the preconcentration process to solutions with initial concentrations of 0.02 to 3 mg L^−1^ of Pb(II). A volume of 5 mL of HNO_3_ 1 mol L^−1^ was used as eluent.

### 2.5. Application of PSf-AA

The developed process for Pb(II) preconcentration was applied in the analysis of water in contact with a handmade glazed clay vessel. To do this, a volume of 100 mL of water that was inside the vessel for 12 h was adjusted to pH 5 with an ionic strength of 0.1 mol L^−1^. The solution was passed through a column previously packed with PSf-AA fiber, following the procedure described in Section 2.4.4.

## 3. Results and Discussion

### 3.1. Elaboration of PSf Polymeric Fibers

**Figure 2:**
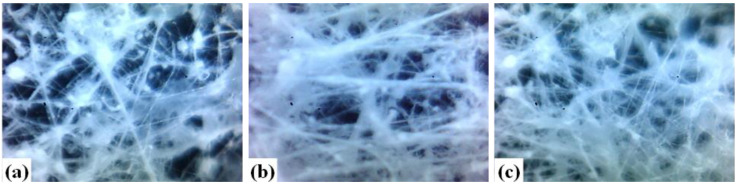
Effect of polymer concentration and distance between collector and airbrush on the morphology of PSf fibers. (**a**) 9%, 10 cm; (**b**) 9%, 15 cm; (**c**) 10%, 15 cm (magnification 400×).

The most pronounced effect of the distance between the airbrush outlet and the collector in the fiber morphology is mainly observed when the distance is minimal (10 cm) (Figure 2a): the solvent does not evaporate, causing the formation of a film or agglomerations (semi-solidified fibrous structures), which are characteristic of the deposition of wet fibers on the collector [34]. On the other hand, at longer distances, the solvent evaporates before reaching the collector, and it decreases the formation of droplets and fibers with a favorable morphology, as shown in Figure 2b. For this reason, 15 cm was chosen as the appropriate distance to manufacture fibers.

**Figure 3:**
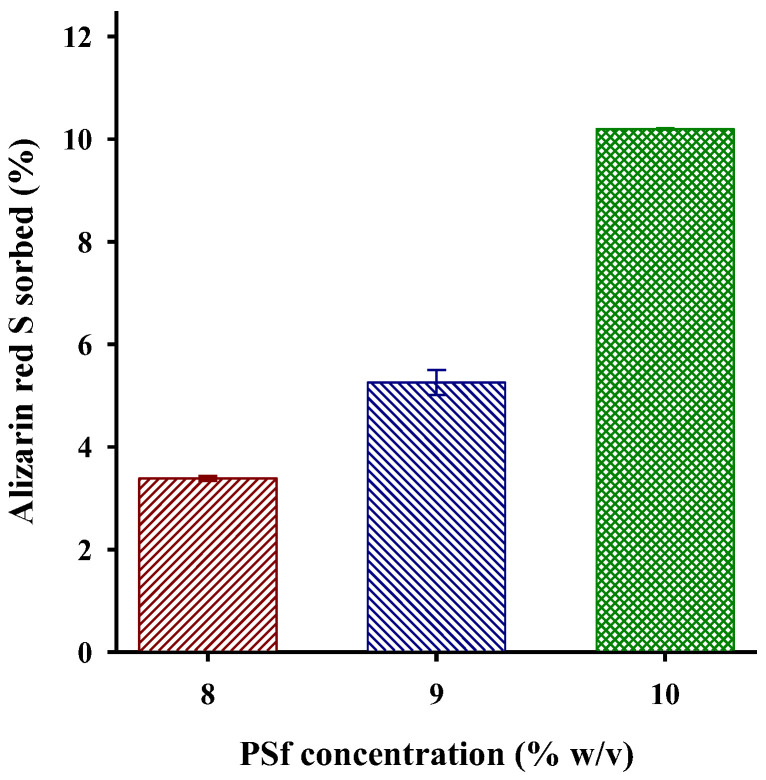
Alizarin red S sorption for quantitative evaluation of effect of PSf concentration on fibers: 10 mg of PSf fibers, 5 mL dye solution 20 mg L^−1^.

Different concentrations of PSf were studied since the concentration of the polymer is closely related to the viscosity of the polymer solution, which has a pronounced effect on the fibers in terms of fiber morphology and diameter [34,35]. As an example, the fibers obtained with 9 and 10% concentrations of PSf are shown (Figure 2b,c, respectively). Fibers obtained with 9% PSf had a greater number of nodules, while, with a concentration of 10% (Figure 2c), a more fibers were obtained. By increasing the concentration to 11%, the solvent evaporated quickly and caused the airbrush to clog, so it was not possible to continue manufacturing fibers with this concentration. Because the 9 and 10% solutions are very close in terms of PSf concentration, the obtained fibers were evaluated quantitatively via the extraction of alizarin red S. Figure 3 shows a higher extraction percentage with the polymeric material containing 10% of PSf. It is attributed to the fact that there are more fibers and, as a consequence, more active sites can retain the dye. The modification of PSf fibers with AA was evaluated with these conditions (10% PSf, distance 15 cm).

### 3.2. Modification of Fibers (Grafting) with AA (PSf-AA)

Since it is difficult to control the number of grafted groups by the process of free radical grafting, the number of active sites attributed to acrylic acid can be determined by quantifying carbonyl groups with TBO. This molecule has a positive charge that can be specifically bonded to carbonyl groups, assuming that they react stoichiometrically in a 1:1 ratio with anionic species [29].

When evaluating the concentration of the initiator solution, it was observed that an increase does not produce fibers with high TBO sorption. This can be attributed to the initiator, since it can start homopolymerization, a secondary reaction in graft polymerization, causing the reaction of the monomer (AA) with itself [36,37]. This causes a decrease in the amount of monomer, damaging the grafting process in the PSf.

Raising the concentration of AA increases the amount of graft in polysulfone, but this also could cause AA to react with itself and form polyacrylate, as has been reported when employing AA concentrations greater than 1.8 mol L^−1^ [36,37]. For this reason, a maximum concentration of 1.6 mol L^−1^ was evaluated and chosen for the grafting process.

By increasing the temperature of the starter solution, the formation of free radicals is favored, thus increasing the amount of active sites in the polymer. However, it is important to consider that the increase in free radicals favors the formation of ending chains causing incomplete polymerization, in addition to converting the solution into a transparent gel, as mentioned above [36,37,38]. To promote the formation of free radicals while avoiding incomplete polymerization, a mild temperature was used in the grafting process (55 °C).

According to experimental design, the best fibers were produced at 55 °C, 1.6 mol L^−1^ of AA, and 0.015 mol L^−1^ of the initiator solution, and had more carbonyl groups determined with the sorption of TBO (0.0087 mmol per g of fiber). These conditions were used for the manufacture of PSf-AA fibers for subsequent experiments.

### 3.3. Fiber Characterization

To corroborate the presence of AA, the characterization of polysulfone (PSf) and polysulfone-acrylic fibers (PSf-AA) was carried out using different techniques. 

The first indicator of possible graft success was the difference in weight measured after the process. The results indicated a weight change that yielded a graft density of 120 μg cm^−2^ (%RSD = 12.9) and a graft yield of 2.36% (%RSD = 0.02). The low percentage of grafting suggests that there was an addition of AA on the surface, but no formation of a homogeneous layer that could cover the fibers.

**Figure 4:**
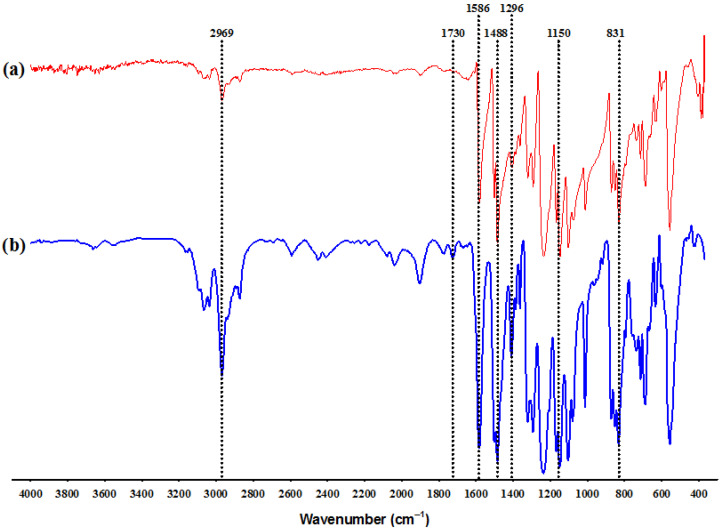
FTIR spectra of (**a**) PSf fibers (10%) and (**b**) PSf fibers grafted with AA (PSf-AA) (grafting conditions: 55 °C, 1.6 mol L^−1^ of AA and 0.015 mol L^−1^ of the initiator solution).

Figure 4 shows the IR spectra obtained from the PSf fiber and the PSf-AA fiber with higher carbonyl content. In the spectrum from Figure 4a, the stretch bands of the sulfone group (O=S=O) can be observed at 1150 and 1296 cm^−1^, and the stretching of the aromatic ring at approximately 1488 and 1586 cm^−1^. Additionally, the para-disubstituted ring C–H out of plane bending vibration is located at 831 cm^−1^. In the spectrum shown in Figure 4b, the appearance of a band around 1730 cm^−1^ corresponding to the AA carbonyl can be observed, which, according to Gancarz et al. [39], shows that acrylic acid is grafted and is not in the form of a film over the polysulfone fibers; this is because the carbonyl signal of the pure polyacrylic acid should be observed between 1703 and 1710 cm^−1^.

In the spectrum, a signal is also observed around 2969 cm^−1^ that could be assigned to the stretching vibration of O–H from carboxylic group and C–H bonds, both from AA [28]. The latter suggests the presence of AA on the surface of the polysulfone fibers in the graft form, as described by Ahmed et al. [40]. 

**Figure 5:**
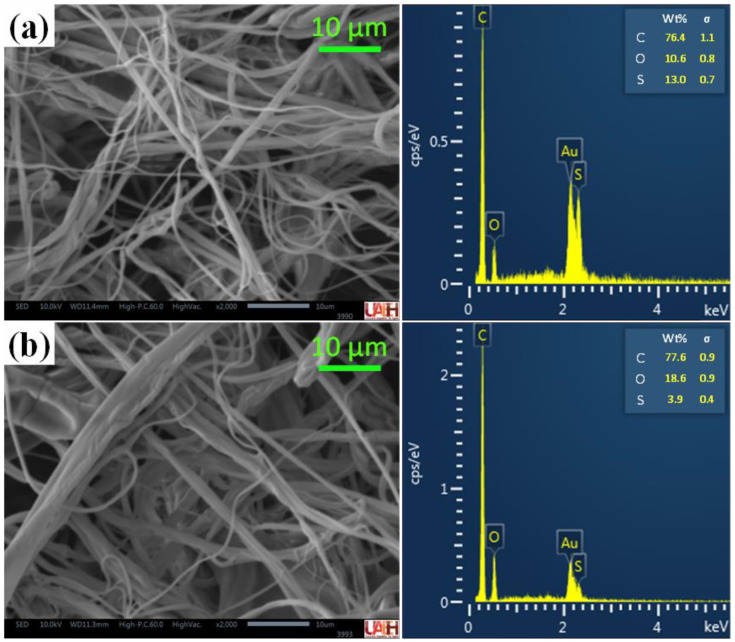
Scanning electron microscopy (SEM) and energy dispersive spectrum (EDS) analysis of (**a**) PSf fibers (10%) and (**b**) PSf-AA fibers (grafting conditions: 55 °C, 1.6 mol L^−1^ of AA, and 0.015 mol L^−1^ of the initiator solution).

After the grafting process, the obtained fibers were observed using SEM to evaluate changes in their morphology. This was confirmed by a difference in the average diameter of the fibers, which increased from 1.82 μm for PSf to 4.66 μm for PSf-AA, derived from the effects which take place in the grafting process. Figure 5 shows the microphotographs obtained and the EDS analysis. In this figure, a difference in carbon and oxygen composition between PSf and PSf-AA fibers is observed. Thus, the carbon percentage increases from 76.4% to 77.6%, and the oxygen percentage from 10.64% to 18.6%, which could corroborate acrylic acid grafting.

### 3.4. Preconcentration of Pb(II) with PSf-AA Fibers

#### 3.4.1. Pb(II) Extraction Process

To develop the pre-concentration process, it is first necessary to sorb the lead in the grafted fibers. For this, they were put in contact with a lead solution in a batch configuration in such a way that a solid–liquid-type extraction was carried out. The extraction of Pb(II) was promoted because this metal has an affinity for the COO^−^ group of acrylic acid. Furthermore, and since acrylic acid from PSf-AA fibers is an ionizable acid, the pH of the solution was varied to find the better conditions for lead extraction. Similarly, other parameters commonly studied in solid–liquid extraction, such as sorbent mass (PSf-AA fibers), contact time, and ionic strength, were evaluated through an experimental design.

**Figure 6:**
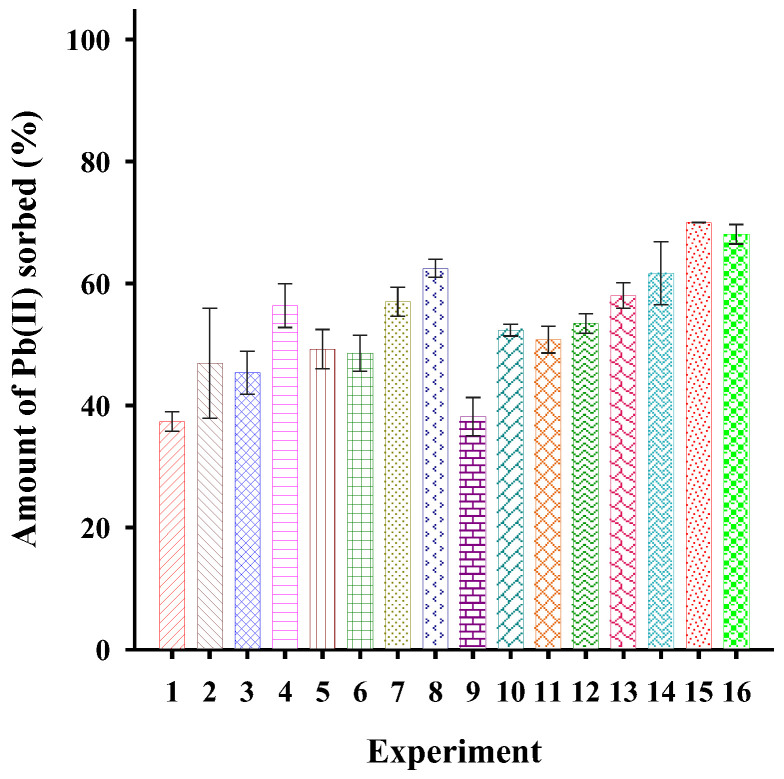
Pb(II) extraction evaluated through a factorial design.

According to the obtained results, it was determined that the conditions to achieve the highest percentage of extraction (70.02%) were those of experiment 15 (pH 5, contact time 90 min, ionic strength 0.1 mol L^−1^ and 20 mg of fibers) (Figure 6).

It was found that the optimal working pH value is related to the AA pK_a_ (4.52 [29]); below this pH value, most of the AA will be protonated, decreasing the interaction with lead and, therefore, its extraction. At pH values of 6 or higher, Pb(II) hydroxo complexes begin to form, competing with free Pb(II) to interact with AA [32,40]. Considering that electrostatic interactions between Pb(II) and AA are better at pH 5 due to their ionic form, this pH was selected for the extraction process.

When evaluating the contact time, better results were found with 90 min; in this time, it was observed that the sorption process reaches equilibrium.

Increasing the ionic strength improves the extraction process due to the electrostatic effect generated by the repulsion of charges that exists between the metal ion and the cation of the used salt, thus causing a greater affinity between the active sites and the metal ions [32].

When the amount of fiber is varied during extraction, it was observed that the more sorbent in the system, the greater the extraction. This is attributed to the increase in the number of active sites. However, in the batch system, the excess mass of fibers causes the films to agglomerate, decreasing the contact area of the fiber with the solution and resulting in a lower extraction percentage.

It is important to mention that the grafting process of AA on PSf fiber is necessary since the unmodified fiber extracts only 13.26% of the Pb(II) in the solution.

With the best conditions previously determined, the Pb(II) sorption capacity of the fibers was evaluated and a maximum sorption capacity of 2.32 mg g^−1^ was found. This value is not as high as those obtained in relevant works previously reported (Table 1), but it has the advantage of having a simple composition of the base polymer while not requiring expensive instrumentation for the preparation of the polysulfone fibers.

Likewise, the process chosen for grafting can be carried out simply and without special equipment. In the same way, the experimental conditions for the extraction of lead from the aqueous solution can be adapted to any laboratory without the use of special reagents, and as will be seen later, the adsorption capacity is not an impediment for the use of fibers in lead preconcentration.

#### 3.4.2. Effect of Interferents on Pb(II) Extraction

**Figure 7:**
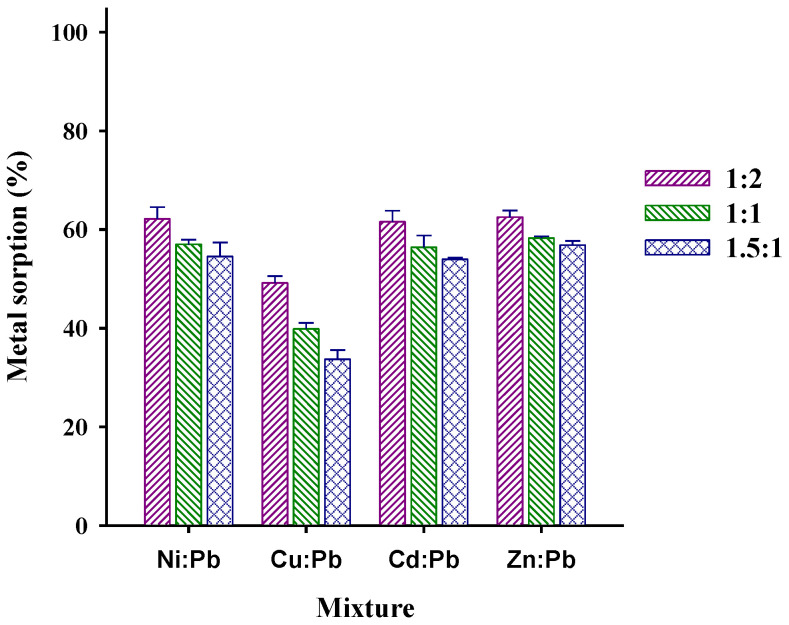
Effect of interferents at different M: Pb(II) proportions on Pb(II) extraction (pH 5, 30 mg of sorbent, 5 mL solution, ionic strength: 0.1 mol L^−1^).

The results obtained from the extraction of Pb(II) in binary mixtures with the selected interferents are shown in Figure 7.

The following order of preference of the material was observed in all proportions of Pb:interferent: Pb(II) > Cu(II) > Cd(II) > Ni(II) > Zn(II). This tendency is similar to that obtained with other modified materials in that the copper extraction percentage is only surpassed by lead when they are extracted from aqueous solutions [15,16]. This may be attributed to variables such as the metal ion hydrated radius, Lewis acid–base interaction, and electronegativity. Thus, the preference for Pb(II) may be attributed to its smaller hydrated radius than the other metal ions (Pb(II): 4.01, Cu(II): 4.19, Cd(II): 4.26, Ni(II): 4.04, Zn(II):4.30 Å), along with being a softer Lewis acid [45]. The smaller radius allows Pb(II) to have a better interaction with the active sites, and since it is a Lewis acid, it will have a preference for bases such as the oxygen present in COO-. Along with the above, it has been reported some divalent metals complexes with polyacrylate, with the formation constant for lead being the largest (log β: 7.1 (Pb(II); 6.6 (Cu(II)); 6.1 (Cd(II)); 5.5 (Ni(II))) [46]. 

The second best-sorbed ion (Cu(II)) has similar characteristics, though it has the third smallest hydrated radius, after Ni(II). It is also a softer Lewis acid, and has higher electronegativity than Ni(II); all this could explain the sorption and competition with Pb(II) [40].

#### 3.4.3. Re-Extraction of Pb(II)

To achieve desorption of Pb(II) from the PSf-AA, two different eluents previously reported in the literature were evaluated: HNO_3_ and EDTA [47]. Both eluents showed a recovery factor of 100%; that is to say, it was found that the milligrams of lead sorbed and eluted were not statistically different. For the following experiments, HNO_3_ was chosen to reduce the matrix effect in atomic absorption analysis.

#### 3.4.4. Preconcentration Process

For this study, column configuration was used. In the preconcentration process, it is first necessary to carry out an extraction in which the Pb(II) solution is passed through a column in which a certain amount of the grafted fiber is packed, and lead is retained. Metal ions are eluted in a smaller volume to be able to preconcentrate them. It is worth highlighting that the water used in the washing step after the re-extraction process did not elute the ions retained in the fibers.

**Figure 8:**
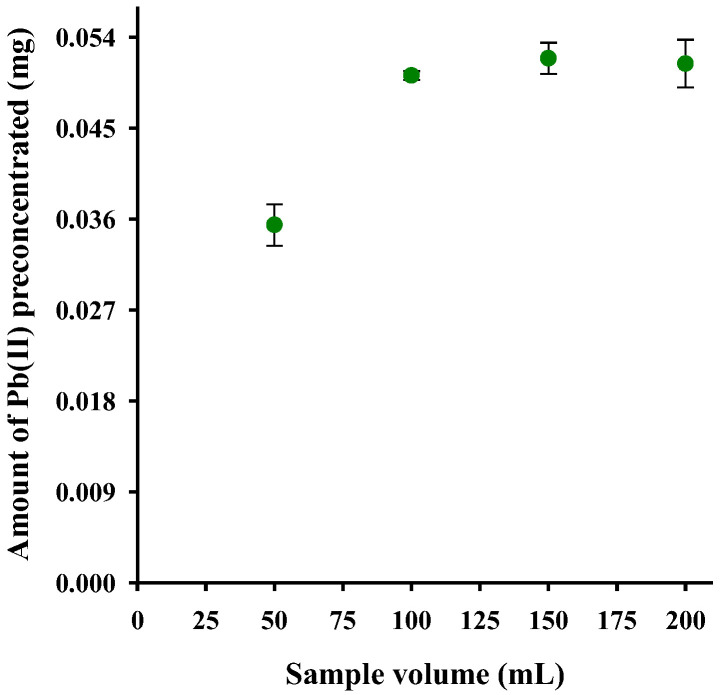
Effect of sample volume on Pb(II) preconcentration (pH 5, 30 mg of sorbent, Pb(II) 1 mg L^−1^, ionic strength: 0.1 mol L^−1^).

The variation of the initial volume of the Pb(II) solution was performed to explore the possibility of analyzing large volumes of solution at low analyte concentrations [12]. The effect of the initial solution volume on Pb(II) sorption was studied by passing through the column packed with Psf-AA fibers, at volumes ranging from 50 to 200 mL. As observed in Figure 8, the amounts of preconcentrated Pb(II) were constant from 100 mL.

**Figure 9:**
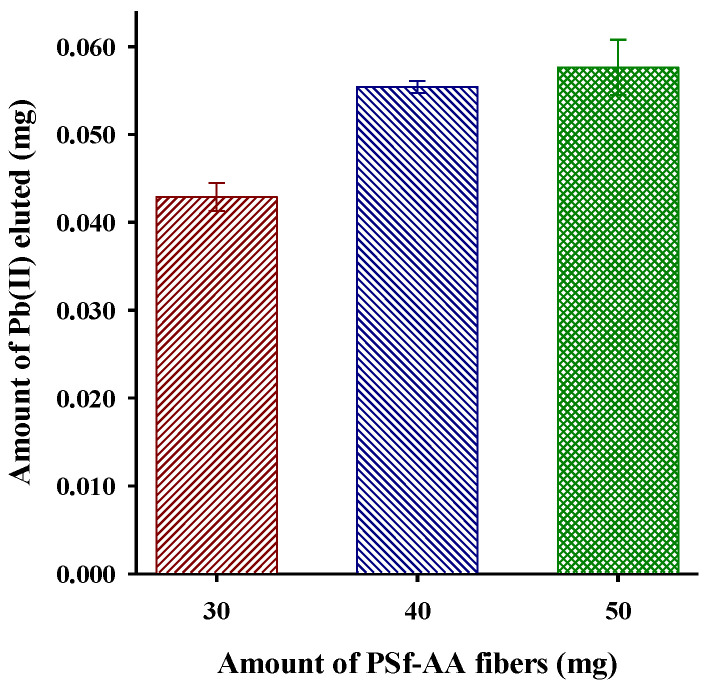
Effect of PSf-AA fiber amount on Pb(II) preconcentration (pH 5, sample volume: 200 mL, Pb(II) 1 mg L^−1^, ionic strength: 0.1 mol L^−1^).

To obtain a higher preconcentration factor, it was decided to conduct a study by increasing the amount of Psf-AA fibers in the column. As a result, having a greater amount of mass, the amount of preconcentrated Pb(II) increased, but from 40 mg of PSf-AA the increase in the preconcentration factor was not significant; this is attributed to saturation of the adsorption sites [12]. The data obtained are shown in Figure 9.

### 3.5. Application of PSf-AA Fibers

To study the potential of the developed sorbent in the preconcentration of Pb(II) in a sample, a calibration curve was made that correlates the initial concentration of lead (analyte) and the concentration obtained after the preconcentration process. Thus, several preconcentration experiments were carried out under the experimental conditions selected in previous sections, but at different initial concentrations of Pb(II), which were close to the permissible limits of this metal ion. The results obtained are given in Table 2.

The method and the analytical figures of the calibration curve were applied to the determination of the amount of Pb (II) in a water sample that was in contact with a glazed clay jar. Preconcentration of Pb(II) was achieved following the procedure described above; the solution was analyzed with FAAS and the absorbance was used to interpolate concentration from the calibration curve.

In a first approach, the determined concentration of Pb (II) in the water sample was 0.2582 mg L^−1^ and was confirmed with the analysis by GFAAS where the initial concentration Pb(II) obtained was 0.2559 mg L^−1^.

Furthermore, three consecutive determinations of leached lead from the glazed jar were performed, and from the data obtained, a paired sample *t*-test was performed (developed method-FAAS vs. GFAAS analysis) (95% confidence, n = 3). This test concluded that there is no significant difference between the Pb(II) concentrations determined using the procedure described above and the determination made using a graphite furnace (t _calculated_ 2.11 < t _critical_ 4.30).

## 4. Conclusions

In this work, polysulfone fibers were produced with a simple blow spinning method and successfully grafted with acrylic acid (PSf-AA). The grafted fibers were used as a sorbent for Pb(II) extraction achieving more than 70% of remotion in a single step; following this process, it was possible to desorb all the removed metal by using HNO_3_. The desorbtion process allowed us to preconcentrate lead and its quantification with FAAS, to reach low concentrations (µg L^−1^). With the developed method, it was possible to quantify leached Pb(II) from a glazed clay jar, finding more than 250 µg L^−1^ in the water sample.

Both the fiber manufacturing process (blow spinning) and the grafting process are simple, fast, low cost, and can be used on a large scale.

Though other methods make possible the quantification of lower concentrations, in this work the fiber fabrication was simpler than other techniques such as electrospinning, and along with the easy grafting process and speed of quantification with FAAS, the developed method is competitive for Pb(II) preconcentration.

## Figures and Tables

**Table 1:** Lead adsorption capacity of fiber-like materials reported in the literature.

Based Fiber Material	Fiber Modifier	Fiber Preparation Technique	Pb(II) Adsorption Capacity (mg g^−1^)	Reference
Polyvinyl alcohol (PVA)	*Malva sylestris* L. (seed biomaterial)	Electrospinning	588.2	[14]
Polyacrylonitrile (PAN)-FeCl_3_	Magnetite (Fe_3_O_4_)	Electrospinning	156.25	[15]
Sodium alginate and polyethylene oxide (PEO)	Graphene oxide	Electrospinning	386.5	[16]
Cellulose acetate (CA) and Fe_3_O_4_	-	Electrospinning	44.05	[41]
Chitosan-TiO_2_	-	Electrospinning	579.10	[42]
Chitosan and polyethylene oxide (PEO)		Electrospinning	214.8	[43]
Polyacrylonitrile (PAN) and Ditizone	NaOH ^1^	Electrospinning	0.016 ^2^	[44]
Polysulfone (PSf)	Acrylic acid	Blow-spinning	2.32	This work

^1^ Used for fiber activation. ^2^ Reported as breakthrough capacity.

**Table 2:** Analytical figures of merit for determination of Pb(II) with the proposed methodology using FAAS (pH 5, sample volume: 200 mL, Pb(II) 1 mg L^−1^, ionic strength: 0.1 mol L^−1^).

Parameters	Values
Linear range (mg L^−1^)	0.008–3.12
Correlation coefficient (R^2^)	0.9999
LOD (mg L^−1^)	0.05
LOQ (mg L^−1^)	0.16
Linear equation (*y = ax + b*)	3.3161x + 0.1347

## Data Availability

Data are contained within the article.
